# Intrinsic wheat lipid composition effects the interfacial and foaming properties of dough liquor

**DOI:** 10.1016/j.foodhyd.2017.08.020

**Published:** 2018-02

**Authors:** Louise J. Salt, Irene González-Thuillier, Gemma Chope, Simon Penson, Paola Tosi, Richard P. Haslam, Peter K. Skeggs, Peter R. Shewry, Peter J. Wilde

**Affiliations:** a Quadram Institute Bioscience, Norwich Research Park, Norwich, Norfolk, NR4 7UA, UK; b Rothamsted Research, West Common, Harpenden, Hertfordshire, AL5 2JQ, UK; c Campden BRI, Station Road, Chipping Campden, Gloucestershire, GL55 6LD, UK; d University of Reading, Whiteknights, Reading, Berkshire, RG6 6AH, UK; e Hovis Limited, The Lord Rank Centre, High Wycombe, Buckinghamshire, HP12 3QS, UK

**Keywords:** Breadmaking, Gas cells, Dough liquor, Foam, Stability, Lipids

## Abstract

Doughs were prepared from a single variety breadmaking flour (cv. Hereward), from three successive harvests (years; 2011, 2012 and 2013). A preparation of the aqueous phase from dough, known as dough liquor (DL), was prepared by ultracentrifugation and its physico-chemical properties were investigated. Surface tension and interfacial rheology, showed that the interface of DL was lipid-dominated and that 2013 DL had a different type of interface to 2011 and 2012 DL. This data was consistent with the improved foam stability observed for 2013 DL and with the types of lipids identified. All foams collapsed quickly, but the most stable foam was from 2013 DL with 89.2% loss in foam, followed by 2011 DL with 91.7% loss and 2012 had the least stable foam with a loss of 92.5% of the foam structure. Glycolipids (DGDG and MGDG) were enriched in 2013 DL, and were also present in DL foam, contributing towards improved stability. Neutral lipids, such as FFAs, were enriched in DL foams contributing towards instability and rapid foam collapse. Baking trials using 2012 and 2013 flour, showed increased loaf volumes and gas bubble diameter in 2013 bread compared to 2012 bread, highlighting the potential impact that surface active polar lipids, enriched in the aqueous phase of dough, could have on improving breadmaking quality.

## Highlights

• During proving, gas bubble stability is determined by the types of lipids adsorbed at the air-water interface of the aqueous phase.
• Both proteins and lipids were active at the air-water interface of dough liquor (DL), but it was dominated by lipids.
• FFAs were enriched in DL foams and were detrimental to foam stability, whilst Polar lipids were enriched in DL.
• Greatest loaf volumes were generated from flour that produced DL with the highest enrichment of polar lipids.

## Graphical abstract

**Figure:**
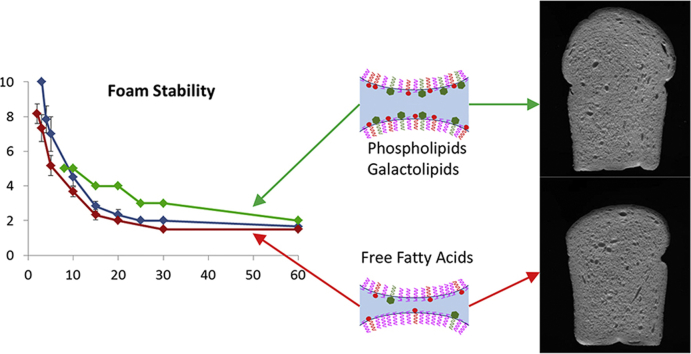

## 1 Introduction

The breadmaking performance of wheat flour is determined by the composition and properties of the grain and the processes used for milling and baking (Cauvain, 2012). Of particular importance is the ability of the flour to form a viscoelastic dough which retains the gas produced during proving and baking to give a loaf with a light porous crumb structure (Chin and Campbell, 2005, Peighambardoust et al., 2010). The physical properties of the dough will depend on various factors, with the amount and quality of the gluten proteins being the most important (D'Ovidio and Masci, 2004, Mills et al., 2012, Shewry et al., 1997). However, the physical properties of the dough will also be affected by other flour components, the dough formulation, including the addition of improvers and surfactants, and the dough mixing process (Cauvain, 2012).

The formation of an elastic gluten network requires shear forces during mixing to allow the proteins to interact and form an elastic network (Belton, 2005, Dobraszczyk and Morgenstern, 2003). The viscoelastic properties of the gluten-starch matrix allow the entrapment of gas cells formed during mixing, which grow during proving leading to the formation of a foam (Campbell & Mougeot, 1999) which is fixed during baking to give a light, porous crumb structure. If the dough is too “strong”, then it will resist the growth of the gas cells, conversely, if the dough is too “weak”, then the network cannot hold the gas cell structure as effectively (Chin & Campbell, 2005), and oven spring (the rapid, final increase in volume during baking) is also reduced (Dobraszczyk & Morgenstern, 2003). Hence, bread quality is determined by gluten strength and dough bubble stability, which have impacts on loaf volume and crumb structure, respectively.

Because the strength of the gluten network influences how gas cells develop, it is not surprising that this is by far the most important factor in controlling bread making quality. Gluten strength is mainly determined by the proportions of individual proteins and their interactions, with one specific protein group, the high molecular weight (HMW) subunits of glutenin, being particularly important (Cauvain, 2012, Chin and Campbell, 2005). However, gluten quality has been estimated to only account for approximately 70% of the variation in overall bread dough functionality (Gupta et al., 1992, MacRitchie, 2016) and attention has focused on the identification of other functional components. In particular, it is likely that whereas gluten plays a key role in gas bubble development, other components are required to confer bubble stability.

Bubble stability determines the extent to which bubbles, created during mixing and proving, coalesce over time. Low levels of coalescence result in the fine texture typical of UK sliced bread, and poor bubble stability leads to a coarser texture and reduced loaf volume. It is clear that surface active components contribute to stabilising bubbles against coalescence, particularly proteins and lipids, but the mechanisms remain unclear (Primo-Martin et al., 2006, Salt et al., 2006, Wilde, 2012). There is therefore a need to elucidate the roles of different wheat components in determining bubble stability and mechanisms of action in order to develop clear targets for improving gas cell stability.

The gas phase in dough is critical for the texture and structure of bread: over 70% of the final loaf volume is made up of gas cells, the size, shape and number of which determines the final texture and structure. Gas cells or bubbles can be created and stabilised in the presence of any amphiphilic molecule, with the molecular structure and physico-chemical properties of the amphiphile (most commonly proteins, surfactants and lipids) determining the foam stability (Wilde, 2012). This stabilising layer is critical during proving of the dough in breadmaking (Campbell & Martin, 2012), as the gas cells come into contact and the risk of coalescence is markedly increased. At this point, the strength of the gluten network no longer controls the stability. Rather, it is the molecular properties of the stabilising layer that control the stability of the bubbles to coalescence, particularly at the end of proving and the start of baking (Hayman et al., 1998, Shimiya and Nakamura, 1997).

Although previous work has focused on the protein and lipid components in dough, their relative contributions have not been defined, as the fragile nature of the dough means that it is very difficult to study the components present at the surface of gas bubbles without destroying the gas cell structure. Several proteins from wheat have been shown to possess surface activity including soluble fractions of gliadins, globulins and albumins (Keller, Orsel, & Hamer, 1997), non-specific lipid transfer proteins (Subirade, Salesse, Marion, & Pezolet, 1995), puroindolines (Biswas et al., 2001, Kooijman et al., 1998, Pauly et al., 2014) and α-amylase/trypsin inhibitors identified in DL foams (Salt, Robertson, Jenkins, Mulholland, & Mills, 2005). However, the consensus is emerging that lipids are the main components controlling bubble stability (Gerits et al., 2014, Sroan and MacRitchie, 2009, Ukai and Urade, 2007).

Wheat flour contains a range of lipids (Pareyt, Finnie, Putseys, & Delcour, 2011), all of which are capable of adsorbing to the surface of the gas bubble, although some are bound up in different structures within the grain and the flour and are effectively not available. Differences in lipid molecular structures will determine the overall bubble stability and the lipid composition of the flour will therefore be critical for dough stability. Bekes et al (Bekes, Zawistowska, Zillman, & Bushuk, 1986). determined lipids in 26 spring wheat flours showing significant correlations between loaf volume and the ratios of neutral lipids to polar lipids and, in particular, of neutral lipids to glycolipids. It has been suggested that phospholipids and glycolipids may promote the formation of protein:lipid complexes during dough-making, through hydrogen bonds and hydrophobic interactions with gliadin and glutenin molecules (Belton, 2005, Dobraszczyk and Morgenstern, 2003). These interactions will in turn result in increased dough strength (as measured by mixing time) and gas retaining capacity and, therefore, in a higher loaf volume and better crumb structure. A role for glycolipids in bread-making was previously suggested by Chung et al (Chung, Pomeranz, & Finney, 1982). based on their structural similarity to bread softeners and surfactants which are commonly added to dough to improve bubble stability. MacRitchie and colleagues (MacRitchie and Gras, 1973, Sroan and MacRitchie, 2009) confirmed that the polar lipid content of dough has a major effect on dough stability and loaf volume and, together with other studies (Gerits et al., 2014, Salt et al., 2006), have shown that the surface properties of dough liquor are dominated by the lipid component. White wheat flour contains a range of polar lipids, including phospholipids (predominantly phosphatidyl choline), galactolipids (predominately monogalactosyldiglycerides (MGDG)) and digalactosyldiglycerides (DGDG)) and lyso-phospholipids (predominately lysophosphatidylcholine (LPC) (Gonzalez-Thuillier et al., 2015), the latter being integral lipids within the starch granules which are released on starch damage (which is affected by milling). Furthermore, lipolytic enzymes can be used to generate novel forms which may have better bubble stabilising properties than the endogenous flour lipids (Gerits, Pareyt, Decamps, & Delcour, 2014).

We report here studies of the role of lipids in gas bubble structure in white flour, using dough liquor and foaming to identify surface-active components. The cultivar Hereward was selected because it was the gold standard for UK bread making wheats for over 15 years, although its protein quality was not outstanding, and grain samples from three successive years (2011, 2012 and 2013) were compared to determine the extent of year to year variation in the amount, composition and properties of the lipids identified as functionally active.

## 2 Materials

Breadmaking wheat, c.v. Hereward was grown under standard agronomic conditions at Rothamsted Research (Harpenden, Hertfordshire UK) in 2011, 2012 and 2013 and milled at Campden BRI (Chipping Campden, Gloucestershire UK), using a Buhler–MLU-202 mill. This gave three break and three reduction fractions, which were combined to give white flour with yields of 79% (2011), 73% (2012) and 77% (2013).

All chemicals and reagents were supplied by Sigma-Aldrich (Poole, Dorset UK) unless otherwise stated.

## 3 Methods

### 3.1 Dough liquor extraction and preparation

Doughs were prepared as previously described by Salt et al. (Salt et al., 2005, Salt et al., 2006). Briefly, doughs were mixed in a Kenwood Chef mixer with a dough hook attachment, mixing for 4 min. Non-yeasted dough (500 g) was prepared using a basic recipe of 305 g flour (61%), 189 g (37.8%) water and 6 g salt (1.2%). The recipe was adjusted for the 2013 flour [318 g flour (63.6%), 175 g water (35%), and 6 g salt (1.2%)] based on the unusually low water absorption of 50.7% (which was determined by Farinograph (to the 600BU Line) using Cereals and Cereal Applications Testing (CCAT) method No. 4).

After dough mixing, 65 g (approximately) dough pieces were weighed into polycarbonate ultracentrifuge bottles (38 × 102 mm) with screw-on titanium caps (Beckman Coulter, item no. 355622), and held at 30 °C (in an incubator) for 90 min in accordance with the common bakery practice in the manufacture of bread by the Chorleywood Bread Process (CBP). The dough was then centrifuged in a pre-warmed (30 °C) fixed-angle rotor (Beckman Coulter, type 45 Ti - item no. 339160) at 200 000 × *g* for 30 min at 30 °C. After ultracentrifugation, the supernatant (dough liquor) was collected, pooled and stirred for 5 min before centrifugation at 48 000 × g for 20 min at 20 °C. The DL separated into three fractions: a TAG-rich lipid pellicle on the top, clarified DL beneath the lipid, and a pellet. The clarified DL was collected using a peristaltic pump, taking care not to cause too much disruption to the lipid layer or the pellet.

### 3.2 DL interfacial properties

A pendant drop technique was used to monitor the surface dilatational moduli of DL. Measurements were taken using an FTA 200 pulsating drop densitometer (First Ten Angstroms, Portsmouth, VA, USA), where a droplet hanging in air, was formed at the tip of a Teflon coated needle (diameter: 1.12 mm) inside a glass cuvette. The needle was connected to a 50 μL glass syringe (Hamilton Company, Reno, NV, USA). Prior to each experiment the syringe and needle were checked for contamination of surfactants by measuring the surface tension of water (72.8 mN/m) for 10 min. The dilatational rheology of DL was then determined by capturing images of a pulsating, 8–15 μl droplet (droplet size was altered depending on DL concentration) that were taken every second for 600 s at approximately 20 °C. The shape of the droplet in each image was analysed by fitting the experimental drop profile to the Young-Lapalce capillary equation to calculate surface tension, volume and specific area. The conductivity of DL (1/10 dilution with ultra-pure water) was measured using a conductivity meter (Radiometer CDM83, Copenhagen Denmark) and a 0.1% NaCl solution to provide a ratio (10% DL: 0.1% NaCl = 2.73 mS: 1 mS), allowing the final salt content of undiluted DL to be calculated (2.73%). For interfacial rheology measurements, DL was diluted with 2.73% NaCl solution to 10%, 1.0%, 0.1% and 0.01% DL.

### 3.3 Foaming

Dough liquor (20 mL) was transferred to a measuring cylinder and was foamed for 15 s, using a mini rotary whisk (Le’ Express, Kitchen Craft, Birmingham UK). The amount of liquid formed underneath the foam (as the foam collapsed) was measured over 60 min.

For determination of lipids enriched in foam, 20 mL dough liquor was transferred to a funnel, with a drainage stopper, and was foamed. After 60 min, the liquid fraction was drained away and the foam was rinsed from the funnel using ultra-pure water.

### 3.4 Lipid extraction

Total non-starch lipids were extracted from white flours, un-foamed DLs and DL foams as described previously (Gonzalez-Thuillier et al., 2015).

*For flours*, non-starch lipids were extracted from flour samples as described by Finnie et al. (Finnie, Jeannotte, & Faubion, 2009) with some modifications. The flour (150 mg) was heated in boiling water (100 °C) for 12 min to inactivate any hydrolytic enzymes (Rocha, Kalo, & Malcata, 2012). Three sequential extractions were then carried out with petroleum ether (PEt), water-saturated butan-1-ol (1:10) (WSB), and propan-2-ol/water (90:10) (IW), with sample to solvent ratios of 1:10, 1:14, and 1:10, respectively. The PEt and WSB extracts were washed by shaking with 1:1 (v/v) 0.88% KCl, centrifugation for 2 min at 650 × g, and recovery of the upper layer to a new tube, in which all three lipid phases were combined.

*For un-foamed DL and DL foam*; lipids were extracted by the Blight and Dyer method with modifications (Bligh and Dyer, 1959, Kates, 1986). Chloroform: methanol (1:2) was added to 1 mL and 4 mL of un-foamed DL and DL foam, respectively in a 2:7.5 ratio. Samples were vortex-mixed and incubated with agitation for 15 min, 250 rpm at room temperature. After 10 min of centrifugation at 650 *g*, the supernatant, containing the dough lipids, was transferred to a new tube. Lipid extraction was repeated using chloroform: methanol: water (1:2:0.8), 3.75 mL and 15 mL for un-foamed DL and DL foam, respectively. The two serial extracts were collected in the same tube. The supernatants were washed with equal parts of chloroform and 0.88% KCL, 1:3.2:3.2 sample: solvent: salt solution ratio. The lower phase was collected in a new tube after centrifugation during 5 min at 650 × *g*. The aqueous phase was re-extracted with 2.5 mL and 10 mL of chloroform for un-foamed DL and DL foam, respectively. For all samples, the combined extracts were evaporated under nitrogen atmosphere at 40 °C, re-suspended in chloroform and filtered (0.45 μm Millex-FH filters, Merck Millipore, Germany), dried under a stream of nitrogen, re-suspended in 1 mL of chloroform, flushed with nitrogen and stored at −80 °C.

### 3.5 Lipid analysis

Quantitative analyses of lipids, including neutral (free fatty acids (FFA), diacylglycerols (DAG) or triacylglycerols (TAG)) and polar (phosphatidylcholine (PC), phosphatidylethanolamine (PE), phosphatidylinositol (PI), phosphatidylglycerol (PG), LPC, DGDG or MGDG) lipids were carried out using electrospray ionization tandem triple quadrupole mass spectrometry (API 4000 QTRAP; Applied Biosystems; ESI-MS/MS) as described previously by González-Thuillier (Gonzalez-Thuillier et al., 2015). The internal standards for polar lipids were supplied by Avanti (Alabama, USA), incorporated as; 8 pmol 13:0-LPC, 0.086 nmol di24:1-PC, 0.080 nmol di14:0-PE, 0.05 nmol di18:0-PI, 0.080 di14:0-PG, 0.03 nmol di18:0-PS and 0.03 nmol di14:0-PA. The standards dissolved in chloroform and different conditions were used for the aqueous samples, 100 μL foam or 25 μL un-foamed DL were combined with chloroform/methanol/300 mM ammonium acetate (300:665:3.5 v/v) to make a final volume of 1 mL.

Neutral lipid molecular species were identified and quantified as described previously (Gonzalez-Thuillier et al., 2015). The amounts of sample used for foamed and un-foamed DL were 100 μL and 25 μL, respectively. The standards were added to the foamed and un-foamed DL samples in the following concentrations 0.607 nmol 15:0-FFA (Sigma Aldrich, St Louis, USA) 0.0857 nmol tri15:0-TAG (Nu-Chek Prep, Minnesota, USA), 0.043 nmol 18:0-20:4-DAG (Sigma Aldrich, St Louis, USA).

### 3.6 Multivariate statistical analyses

Principal Component Analysis (PCA) was generated from full datasets for the individual molecular species of the major lipid groups of white flour, DL and DL foam from 2011, 2012 and 2013. Multivariate statistical analysis software (SIMCA-P, version 14, Umetrics, Umea) was used with unit variance scaling to compensate for differential concentrations of each lipid species in the flour, DL and Foam.

### 3.7 Protein determination

The protein content of the dough liquor was determined by infrared (IR)-based protein quantitation, using a bench-top Direct Detect^®^ infrared spectrometer (Merk Millipore, Herts, UK). In brief, 2.0 μL of sample (diluted to 1:10 using 2.73% NaCl solution) was transferred onto a hydrophilic polytetrafluorethylene (PTFE) membrane (which is transparent in mid-IR regions used for protein analysis), on a sample card, and air-dried (using the heater in the spectrometer) before use. Protein contents were calculated against a BSA standard curve using a simple univariate (Beer-Lambert) analysis applied by the software of the spectrometer (which relies on integration of the Amide I band).

### 3.8 Test baking

Test baking of the 2012 and 2013 flours and a control flour (Centurion, a commercially-available bread-making flour (Whitworth Bros Ltd)), was carried out using a standardised protocol based on the Chorleywood Bread Process. A lean recipe was used, with 15 g salt (1.5%), 0.1 g ascorbic acid (0.01%), 0.014 g fungal alpha amylase (0.0014%), 22.5 g yeast (2.25%), added to 1 kg flour and water added according to the water absorption (determined by Brabender Farinograph to the 600BU line]. Doughs prepared with gluten fortification (up to 11%) to match that of the control and were mixed using a Morton mixer to a work input of 11 Wh/kg and to a final dough temperature of 30.5 ± 1 °C. The doughs were divided into 465 g pieces and were proved to a height of 10 cm at 40 °C in humid conditions to prevent skinning. Proven dough was baked in a direct gas-fired reel oven at 235 °C for 25 min resulting in single piece 400 g unlidded loaves. Loaves were assessed for height, volume and crumb structure (using a C-Cell instrument, Calibre Control International, UK).

## 4 Results

### 4.1 Flour lipid composition and properties

Total lipids were extracted from flour identified and quantified by ESI-MS-MS. The lipid classes identified were, (a) neutral lipids: including free fatty acids (FFA), diacylglycerol (DAG), triacylglycerol (TAG); (b) galactolipids: monogalactosyl diglycerol (MGDG) and digalactosyl diglycerol (DGDG); and (c) phospholipids: phosphatidyl choline (PC), lysophosphatiyl choline (LPC), phosphatidylinositol (PI), phosphatidylserine (PS), phosphatidylethanolamine (PE), phosphatidylglycerol (PG), phosphatidic acid (PA).

The lipid composition of the flours differed, and neutral lipids were most abundant and galactolipids were least abundant for all three years (Fig. 4). The 2013 flour had the highest amounts of neutral lipids, 9274 nmol/g flour (Fig. 4), accounting for 73 mol % of total lipids; containing the highest amount of TAGs (7967 nmol/g flour), DAGs (609 nmol/g flour), and the lowest amount of FFAs (697 nmol/g flour) (Table 1). Followed by 2012 flour, containing 4984 nmol/g flour neutral lipids (Fig. 4), accounting for 89 mol % of total lipids; containing 2533 nmol/g flour TAGs, 609 nmol/g flour DAGs and 6974 nmol/g flour FFA (Table 1). The 2011 flour had the lowest amounts of neutral lipids (3331 nmol/g flour) (Fig. 4), accounting for 65 mol % of the total lipids. The flour from 2011 had the, highest amount of FFAs (2503 nmol/g flour) and the least amount of TAGs (698 nmol/g flour) and DAGs (130 nmol/g flour) (Table 1).

**Table 1:** Total lipid composition in White Flour, DL and Foam on three different years (2011,2012 and 2013) represented as nmol/g flour. The mean is the average of at least three biological replicates. Each lipid class represents the sum of all molecular species detected by mass spectrometry for each class.

Lipid class	White Flour 2011 (nmol/g flour)		White Flour 2012 (nmol/g flour)		White Flour 2013 (nmol/g flour)		DL 2011 (nmol/g flour)		DL 2012 (nmol/g flour)		DL 2013 (nmol/g flour)		Foam 2011 (nmol/g flour)		Foam 2012 (nmol/g flour)		Foam 2013 (nmol/g flour)	
	MEAN	SE	MEAN	SE	MEAN	SE	MEAN	SE	MEAN	SE	MEAN	SE	MEAN	SE	MEAN	SE	MEAN	SE
TAG	698.4	22.9	2532.9	99.6	7967.4	207.5	75.8	4.8	77.4	4.1	103.8	1.1	120.5	10.1	117.0	8.4	432.5	50.5
FFA	2502.6	295.7	1897.8	42.0	697.4	30.8	209.6	23.0	122.3	13.9	102.3	8.8	1189.0	295.8	373.2	8.7	468.9	65.7
DAG	130.3	7.7	553.3	19.6	609.0	23.9	16.7	3.1	34.4	5.4	33.0	6.1	39.2	10.4	45.9	11.6	291.0	9.3
DGDG	381.5	12.9	531.7	11.2	783.7	11.7	16.1	2.0	46.8	11.5	742.8	53.4	12.5	7.7	11.3	4.3	269.2	94.0
MGDG	126.6	4.0	206.7	2.9	316.1	4.9	7.9	1.1	29.8	7.7	363.7	8.9	7.5	3.7	9.5	2.6	134.7	53.8
LPC	1145.2	24.3	1612.4	41.0	1335.1	37.7	7.9	2.6	10.4	4.0	35.5	0.5	8.5	1.7	1.7	0.2	31.1	3.1
PC	51.0	3.0	205.7	7.8	524.7	29.3	3.0	0.3	32.6	10.6	228.0	63.6	6.3	2.6	24.6	11.7	366.2	228.1
PG	1.6	0.1	1.6	0.2	7.3	0.4	0.2	0.0	0.4	0.1	3.7	0.3	0.4	0.2	0.3	0.1	1.0	0.2
PE	2.7	0.2	9.6	0.7	29.8	1.7	0.2	0.0	1.3	0.7	18.5	2.5	0.1	0.0	0.4	0.2	1.5	0.7
PI	44.6	1.0	66.2	5.2	373.8	11.2	0.4	0.0	0.9	0.1	6.4	0.9	0.7	0.2	1.9	1.0	8.2	2.1
PS	12.7	1.1	9.3	0.6	20.7	0.8	0.1	0.0	0.1	0.0	0.7	0.1	0.6	0.3	0.4	0.1	0.3	0.0
PA	3.1	0.2	4.7	0.8	47.5	4.9	0.1	0.1	1.0	0.5	3.6	1.1	2.1	0.9	0.7	0.5	1.2	1.2
Total lipids	5100.2	304.6	7632.0	126.8	12712.5	296.6	337.9	25.4	357.3	43.9	1641.9	106.1	1387.5	310.2	586.9	45.4	2005.9	491.3

Galactolipids were present in the biggest quantities in 2013 flour (1100 nmol/g flour), followed by 2012 flour (738 nmol/g flour), and the lowest amounts were found in 2011 flour (508 nmol/g flour) (Fig. 4), accounting for 9 mol%, 10 mol% and 10 mol% total lipids respectively. In terms of specific lipid classes, 2013 flour contained the highest amounts of DGDG (784 nmol/g flour) and MGDG (316 nmol/g flour); compared to 2012 flour which had 532 nmol/g flour DGDG and 127 nmol/g flour MGDG. The 2011 flour had the least amount of galactolipids, comprising 381 nmol/g flour DGDG and 127 nmol/g flour MDGDG (Table 1).

The 2013 flour had the highest levels of phospholipids (2339 nmol/g flour), compared to 2012 (1910 nmol/g flour) and 2011 flours (1261 nmol/g flour) (Fig. 4), accounting for 18 mol%, 25 mol% and 25 mol% of total lipids. The largest contribution towards the phospholipids was from LPC where 2012 flour had the highest amount (1612 nmol/g flour), compared to 2013 flour (1335 nmol/g flour) and the least amount in 2011 flour (1145 nmol/g flour). Phosphatidylcholine (525 nmol/g flour) and PI (374 nmol/g flour) made significant contributions to the total amounts of phospholipids in 2013 flour. The remaining phospholipids were much less abundant for all three samples.

These differences could be related to environmental effects on grain composition and/or differences in milling but we consider that they are probably unlikely to result from lipid breakdown during flour storage as all flours were stored at −20 °C and our investigations showed that lipid breakdown was negligible under these conditions (results not shown). It is notable that the samples also differed in water absorption, which was lower in 2013 requiring the addition of less water for dough mixing. Although water absorption is generally determined by the extent of starch damage during milling, the reason for the difference was not determined in the present study.

### 4.2 Surface properties of dough liquor

**Fig. 1:**
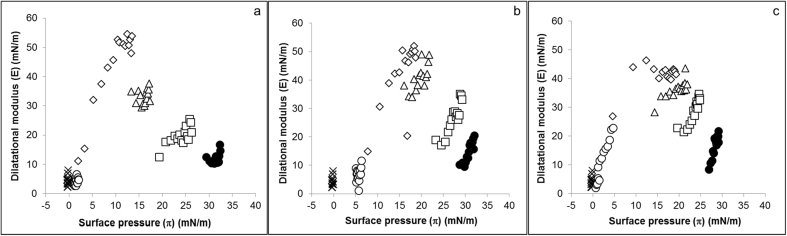
Surface dilatational rheology of DL from 2011 (a), 2012 (b) and 2013 (c). Undiluted DL (●) was diluted to 0.01% (◯), 0.1% (◇), 1% (△), 10% (□); DL measurements were compared to ultrapure water (✕).

**Fig. 2:**
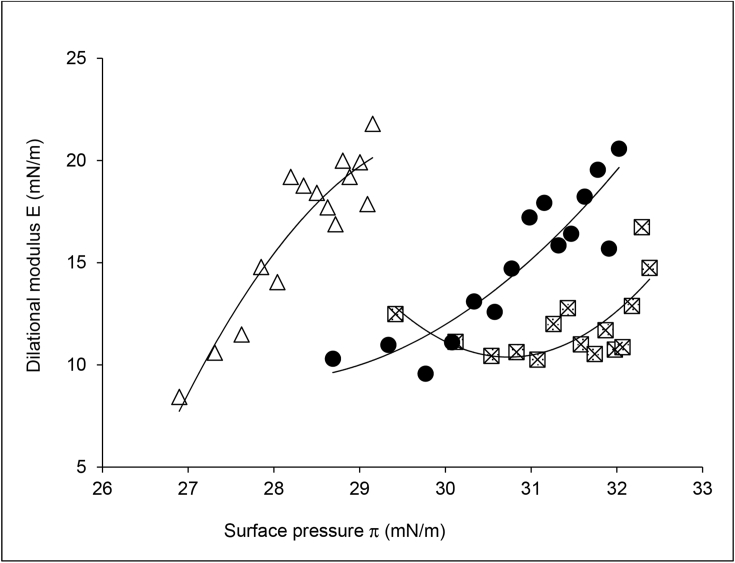
Surface dilatational rheology of 100% DL from 2011(⊠), 2012 (●) and 2013 (△) flours as a function of the surface pressure.

The surface pressure (π) of the samples was calculated by subtracting the mean surface tension of the samples from the surface tension of water (72.8 mN/m at 20 °C, but can vary with temperature) measured at the time of analysis. Averaged surface dilatational elastic modulus (Ε), obtained from 15 intervals over 600 s, was plotted against surface pressure (π) to indicate the types of molecules adsorbed at the air/water interface of DL at a range of concentrations (0.01%, 0.1%, 1.0%, 10% and 100%). The resulting values of Ε for 2011 (Fig. 1a), 2012 (Fig. 1b) and 2013 (Fig. 1c) showed that, for all samples, the surface of DL was dominated by proteins at lower DL concentrations (0.1% and 1.0% DL). However, as the concentration of DL was increased, so did the lipid content which resulted in increased surface pressure and a decrease in Ε to values typical of interfaces dominated by lipids, as previously described by Salt et al. (Salt et al., 2006). Dough liquor, diluted to 0.01% for all samples, showed similar interfacial rheological properties to water and was too dilute to obtain any information about the DL interface. Dough liquors from 2011 to 2012 flours showed similar interfacial rheological properties, however, some small changes were observed for the 2013 flour (Fig. 1c) at 100% DL. The surface pressure (π) of DL from 2011 to 2012 flours ranged between 30 and 35 mN/m (Fig. 1a and b) but for 2013 (Fig. 1c), π was lower at a range of 25–30 mN/m. To demonstrate the differences between the years in more details, the surface elasticity (Ε) was presented as a function of surface pressure (π) for undiluted DL from 2011, 2012 and 2013 flours on the same graph (Fig. 2).

In terms of elapsed time during each experiment, the earliest adsorption time equates to the lowest values of π, and as adsorption continues, π increases for DL from all three growing years. The initial increase in π relates to the development of the air: water interface by the migration of surface active molecules in the DL, such as proteins, to the interface followed by their rearrangement and interaction however, the π values obtained are higher than would be expected from protein alone (<24 mN m^−1^), and are normally associated with interfaces occupied by low molecular weight surfactants or lipids. Therefore it is likely that such large increases in π are the result of small amounts of lipid continually adsorbing into the interface disrupting any interfacial protein networks, as previously shown by Salt et al. (Salt et al., 2006). Fig. 2 shows that the 2011 and 2012 flours had a similar range of values to each other, although the trends were slightly different, with both being distinctly different to the 2013 flour. The higher π values for the 2011 and 2012 flours would indicate a greater emphasis of surfactants or lipids on their surface properties compared to the 2013 flour. The difference in trends between the 2011 and 2012 could indicate that kinetic changes in surface composition or molecular interactions over the course of the experiment are slightly different between these two samples.

All samples displayed relatively weak elastic properties (Fig. 2), with low Ε values indicating a surface that is strongly influenced by the presence of lipids, as it is known that even small amounts of lipids can have a significant effect on surface rheology (Wilde, 2000). Dough liquor from 2011 flour produced the least elastic interface; where Ε was approximately 10–12 mN/m for most of the study, only rising towards 15 mN/m towards the end of the experiment. The Ε values for the 2012 and 2013 DLs were over a similar range and trend, although 2013 DL showed a more rapid rise in Ε during the earlier stages of the experiment, i.e. at the lower π values. The Ε values became similar between 2012 and 2013 towards the end of the experiment. If the interfacial composition of 2 different samples was the same, but the kinetics of adsorption was different, then the data presented in Fig. 2 would overlay between the 2 samples. This is because plotting the data as a function of π normalises for any differences in adsorption kinetics (Ridout, Mackie, & Wilde, 2004). Therefore, the distinct differences observed between the samples in Fig. 2 clearly demonstrate that the DL from 2013 flour had a different surface composition to DL from 2011 to 2012 flours, possibly due to there being more surface-active protein in the 2013 DL (Supplementary Fig. 1.) available for adsorption.

### 4.3 Dough liquor foam stability

Foams were generated from 20 mL DL using a rotary whisk to determine their stability and relate these properties to the stability of bubbles in bread dough. Observations by the authors and others have shown that whole DL extracted from unmodified flour does not foam (data not shown). This is thought to be due to the presence of neutral lipids such as triglycerides which have a detrimental effect on foam stability and loaf volume (Sroan & MacRitchie, 2009). However, the lipid pellicle was excluded from DL during preparation (Section 3.1) so that most of the triglycerides were also excluded, allowing the shearing power of the whisk (traditionally used for producing milk foams for coffee) to generate foam from DL.

**Fig. 3:**
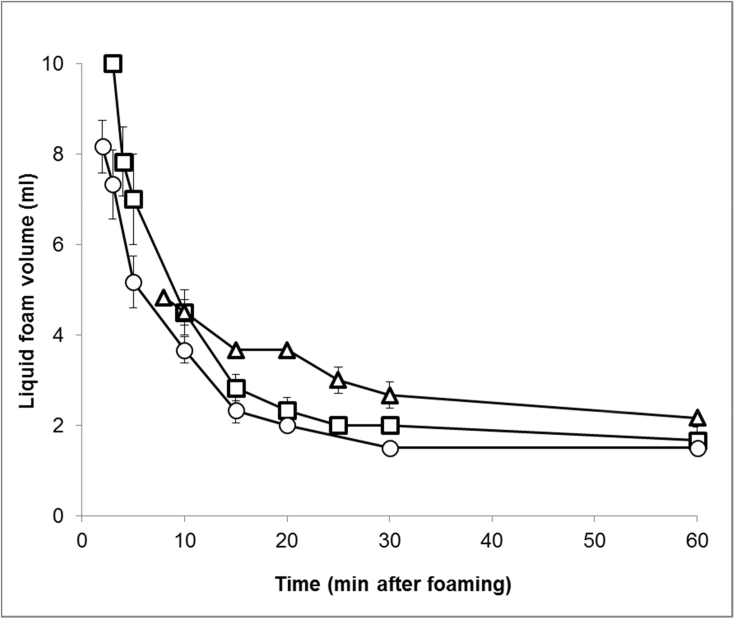
Foaming properties of dough liquor from 2011(□), 2012 (◯) and 2013 (△) flours.

Although the DLs foamed well, the foams were unstable and collapsed quickly with the least stable foam generated from 2012 DL, and the most stable from 2013 DL (Fig. 3). Foam volume measurements were taken when a distinct border was observed between the foam and the drained DL underneath the foam (the foam that had collapsed). For 2012, this border appeared 2 min after foaming where the foam volume was 8.2 mL (59% reduction), which decreased rapidly to 3.7 mL (81.7% reduction) at 10 min after foaming and collapsed further reaching 2.3 mL at 15 min after foaming (88.3% reduction). By 30 min, the foam volume had collapsed further to 1.5 mL and remained at this volume until 60 min after foaming resulting in a 92.5% loss of foam structure. The foam generated from 2011 DL behaved in a similar way to 2012 DL but was slightly more stable with a slower foam drainage rate. The border between the foam and the drained DL was visible slightly later, at 3 min after foaming, with a foam volume of 10.0 mL (50% reduction), which again drained quickly so that there was a 77.5% reduction in foam volume (4.5 mL foam) at 10 min after foaming. After 15 min the foam volume had fallen to 2.8 mL – a reduction of 85.8%, which collapsed further to 2.0 mL (90% reduction) after 30 min and to 1.5 mL at 60 min resulting in a 91.7% loss of foam structure. The DL from 2013 flour gave the most stable foam with slower drainage than the foams from 2011 to 2012 DLs. Also, the border between the foam and the DL was not visible until 8 min after foaming when the foam volume was 4.8 mL (75.8% reduction) which slightly decreased to 4.5 mL (77.5% reduction) after 10 min, and after 15 min the foam had depleted to 3.7 mL (81.7%). At 30 min after foaming, the foam volume had further decreased to 2.7 mL (86.7%), and then after 60 min reached 2.2 mL resulting in an 89.2% loss of foam structure.

### 4.4 Lipid analysis of dough liquor and foam

Total lipids were extracted from DL and DL foam fractions, to identify which were enriched in DL foams, and compared to those extracted from white flours. A total of 85 molecular species were identified and quantified by ESI-MS-MS and variation was observed both between sample type and year of harvest. Lipid analysis data showed that DLs and foams from 2011 to 2012 contained more neutral lipids than galactolipids or phospholipids, except for 2013 DL, which had more galactolipids and phospholipids than neutral lipids and more polar lipids enriched in its foam than 2011 and 2012.

**Fig. 4:**
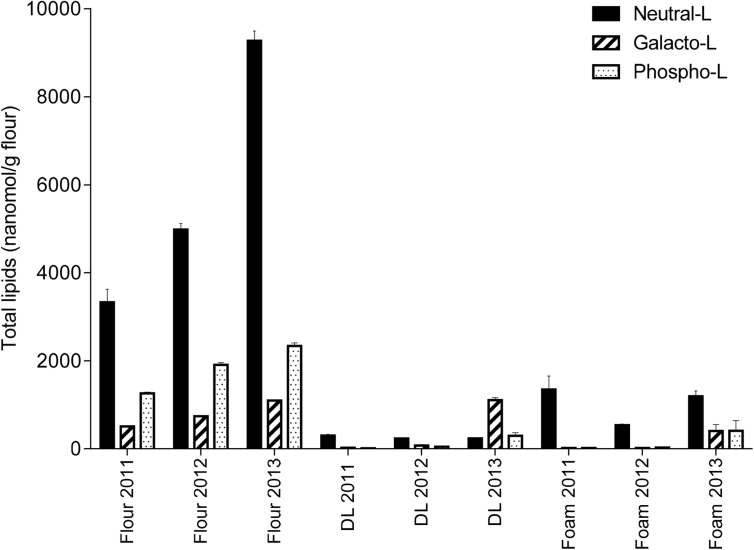
Lipid group distribution in flour, DL and DL foam. Neutral lipids (including FFA, DAG and TAG), galactolipids (MGDG and DGDG) and phospholipids (including LPC, PC, PG, PS, PE, PA and PI).

The 2011 DL had the highest amounts of neutral lipids (302 nmol/g flour) compared to 2012 DL (234 nmol/g flour) and 2013 (239 nmol/g flour) (Fig. 4), accounting for 89 mol %, 65 mol % and 14 mol % of total lipids respectively. The neutral lipid content of DL foam was greatest for 2011 where 1349 nmol/g flour was determined, an enrichment of 78%; followed by an 80% enrichment in 2013 foam (1192 nmol/g flour); 2012 DL contained the lowest amounts of neutral lipids, resulting in a lesser enrichment of 56% (536 nmol/g flour) in its foam. Small quantities of glycolipids were determined for 2011 DL (24 nmol/g flour) and 2012 DL (77 nmol/g flour) accounting for only 0.5 mol% and 1 mol% total DL lipids respectively (Fig. 4). However, 2013 DL had much higher levels of glycolipids; 1106 nmol/g flour, similar to the amounts present in 2013 flour and accounting for 9 mol% of total DL lipids. Galactolipids were present in DL foams but they were not enriched like the neutral lipids were. However, 2013 foam contained the highest amounts of galactolipids (404 nmol/g flour) and phospholipids (410 nmol/g flour), accounting for 40 mol% of total foam lipids for both groups.

The amounts of classes of lipids varied widely among the samples (Table 1). The neutral lipids were most abundant group in the flours. Free fatty acids had the lowest values for 2013 DL (102 nmol/g flour) and 2012 DL (122 nmol/g flour) and were highest in 2011 DL (210 nmol/g flour). The FFAs were enriched in the foams for the three years (2011: 1189 nmol/g flour; 2012: 373 nmol/g flour; 2013: 469 nmol/g flour), where 2011 had the greatest enrichment (82%). The 2013 DL had the lowest amounts of DAG (33 nmol/g flour), compared to 2011 and 2012 DL, but DAG was enriched in 2013 foam (291 nmol/g flour; 87% enrichment), the highest levels out of the three years. Although TAG was identified in the DLs and DL foams, we intentionally excluded the lipid pellicle on the surface of DL to exclude most of the TAG, which would have affected surface tension and surface rheology measurements and obscure the effect of other surface-active lipids. Our justification for this is that during baking, TAG droplets would be entrapped in the starch-gluten matrix, and would not be able to diffuse towards the gas bubble surface and thus are likely to have less of an impact than the polar lipids. Any TAG present in the DL would therefore arise from contamination of the capillary tubing used to extract the clarified DL after a secondary centrifugation step (see section 3.1). We therefore do not discuss the data for TAGs.

Fig. 4 shows that galactolipids were less abundant in the flour than the neutral lipids, so generally lower amounts of DGDG and MGDG were found in DL and DL foams. However, the 2013 DL and foam was an exception, with significantly higher amounts of galactolipids than 2011 and 2012. Table 1 shows that the 2011 DL had the lowest amount of DGDG (16 nmol/g flour; 5 mol% of total lipids), compared to 2012 DL (47 nmol/g flour; 13 mol% of total lipids) and 2013 DL had the highest levels (742 nmol/g flour; 45 mol% of total lipids); similar to the amount in 2013 flour. The 2013 foam had the highest amounts of DGDG (269 nmol/g flour; 13 mol% of total lipids). The highest amount of MGDG (364 nmol/g flour; 22 mol% of total lipids) was found in 2013 DL and 2013 foam also had the highest levels (135 nmol/g flour; 7 mol% of total lipids).

Phospholipids were present in higher quantities in the flour than glycolipids, but they were not as abundant as the neutral lipids, so, like the glycolipids, less were available in DL for enrichment in the foam. The 2013 DL had the greatest amount of PC, 228 nmol/g flour which enriched in the foam by 38%–366 nmol/g flour. Also, PI was also enriched in 2013 foam, but not to the same extent as PC. The PI content of 2013 DL was 6 nmol/g flour and increased to 8 nmol/g flour in the foam with an enrichment of 25%. Foamed samples from 2011 DL and 2012 DL also showed an enrichment of PI, PS and PA, although contributions to the lipid group are relatively small.

**Fig. 5:**
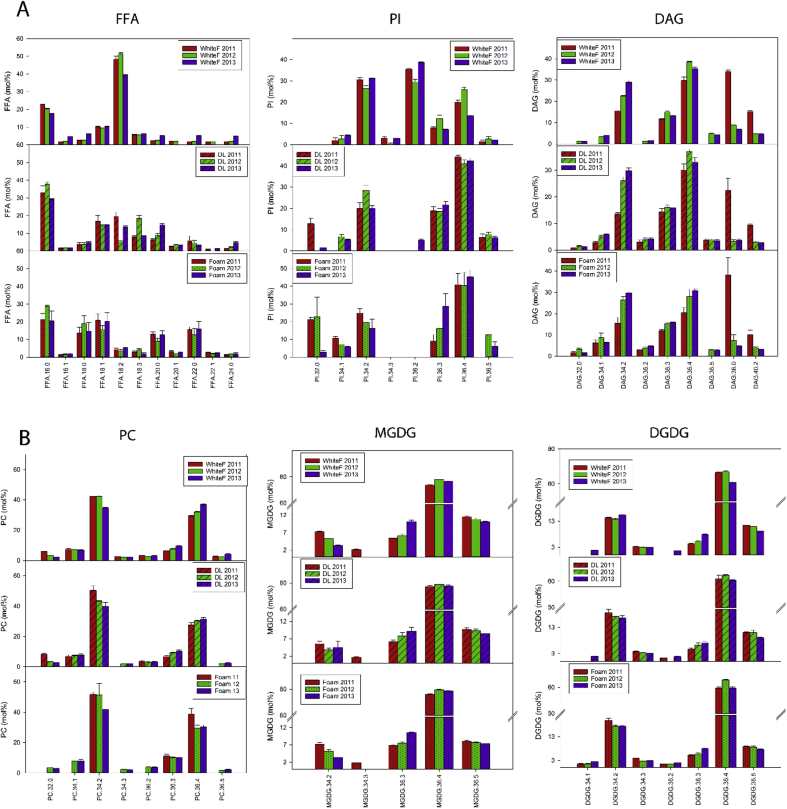
Proportions of molecular species in different lipid classes (by chain length) identified from flour, DL and DL foam. A) FFA, PI and DAG. B) PC, MGDG, DGDG. Expressed as mol% of total lipid content.

In terms of specific molecular species Fig. 5, shows the proportion of the different molecular species as a percentage of lipid content. Some differences were observed in the degree of enrichment in some classes of lipids. These differences were highest for FFA; the 18:2 species was predominant in white flours, accounting for up to 51 mol% of total FFAs in 2012 samples. However, the proportion of this species decreased dramatically in DL foams; by more than 12-fold in the 2012 DL foam. By contrast, other saturated and monounsaturated FFAs were enriched in DL, especially in the foams. That was also the case for 18:1 which was increased by 11% in 2011 foams compared to white flour. The FFA20:0 reached levels of 14% in 2013 DL and 13% in 2013 foam, whereas in the 2013 flour it represented only 5% of the total FFA. The enrichment of the FFA22:0 was even greater in the 2011 foam compared to the white flour with a 9-fold increase. Within the phospholipids, the PI also differed between white flour, DL and foam. For example, the proportion of the species PI34:2, and in particular, PI36:2 were reduced in foams and DLs, falling below the detection limits for the MS analysis. On the other hand, PI36:3 and PI36:4 were enriched in foams and DLs, being 4 and 3 times higher, respectively, in white flour compared to foams in 2013.

The proportions of DAG38:0 and DAG40:2 were greater in the 2011 white flour, DL and foam samples compared to the other years but there were no differences between sample types. Despite the increases in DGDG, MGDG and PC in foams, especially in 2013, there were no differences in the proportions of individual molecular species in these polar lipid classes between the samples.

### 4.5 Multivariate analyses

**Fig. 6:**
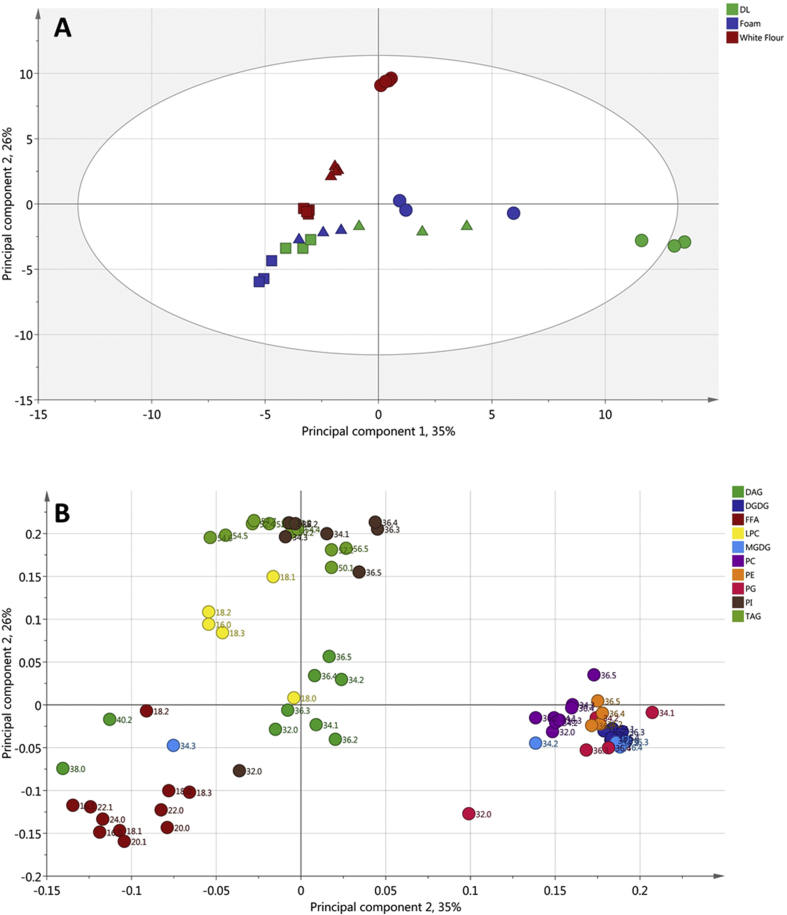
Principal Component Analysis (PCA) of lipid composition. PC1 (35%) vs PC2 (26%). (A), PCA scores plot showing white flour (red), DL (green) and foam (blue) samples from 2011 (▪), 2012 (▲) and 2013 (●). (B): PCA loading plot showing DAG, DGDG, FFA, LPC,MGDG, PC, PE, PG, PI and TAG lipid classes and their molecular species. (For interpretation of the references to colour in this figure legend, the reader is referred to the web version of this article.)

The molecular species of the major lipid groups, in the different samples, were compared by PCA (Fig. 6). The first three Principal Components explained 76% of the total variance. The PCA showed sample distribution according to lipid composition. Noticeably, samples were distributed in a gradient according to the year when they were harvested, and some sample types were separated from the rest due to differences in lipid composition (Fig. 6). For instance, 2013 white flour showed a positive score in Principal Component 2 (PC2) given by an enrichment in TAG and PI molecules, differentiating from the other years and the rest of the samples (Fig. 6A, and Supplementary Fig. S2A and S3). Flours from 2011 to 2012, as well as DL and DL foam from the same two years, had a negative score along the PC1 axis, due to higher amounts of FFA and lower amounts of glycolipids and PL compared to samples from 2013. A group for 2013 DL was plotted on the far-right side of the chart (Fig. 6A), and can be explained by a positive score in PC1 due to an enrichment of glycolipids (specifically DGDG and MGDG) and PL (including PC, PG and PE), as well as lower amounts of FFAs (supplementary Fig. S2 B and C). However, the group was positioned in the negative part of PC2 due to lower amounts of TAG and PL (Fig. 6A, Supplementary Fig. S2A and S3). Differences in TAG in DL and DL foams are down to DL preparation, described in section 3.1, thus comparing TAG levels in DL and DL foam is not possible for this work. A high positive score from 2013 foam, in the PC3 axis resulted in a distinct group present in the top part of the plot, due to higher levels of DAG and lower levels of FFA and LPC (Supplementary Fig. S1A and B). The differences in lipid composition, explains why DL 2013 was showing better foaming properties than 2011 and 2012 DL. White flour from 2011 was grouped at the bottom of the plot showing opposite characteristics in terms of NL and LPC composition in 2013 foam (Supplementary Fig. S1B).

### 4.6 Test baking

**Fig. 7:**
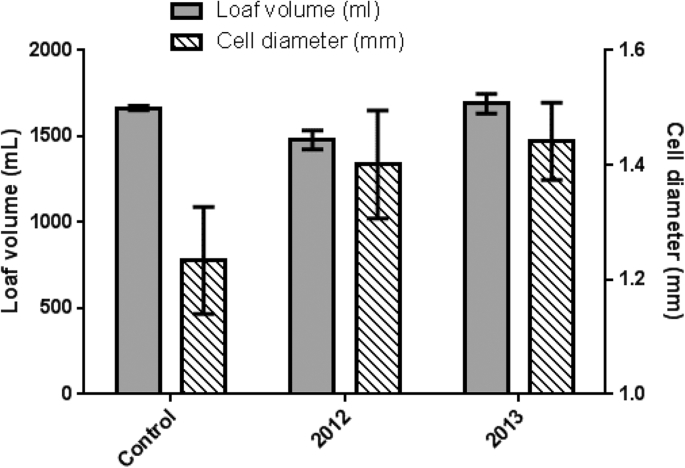
Loaf volume and gas cell diameter measured on loaves made during the preliminary baking trials.

Test baking was carried out on the flours from 2012 to 2013, with the addition of gluten to equalise the protein content of both flours with the control. The data clearly show that the loaves baked from 2013 flour had higher loaf volumes (2012 = 1479 mL; 2013 = 1690 mL) and the diameter of the gas cells was also greater compared to loaves baked with the 2012 flour (2012 = 1.40 mm; 2013 = 1.44 mm) and the control loaves (1.23 mm), which were not fortified with gluten (Fig. 7). The dough formulations had been adjusted to match the protein content, and account for the water holding capacity of the test and control flours. This would tend give the doughs more similar rheological properties to each other to optimise for air incorporation during mixing and proving. However, the rheology of the doughs are unlikely to be identical, as the gluten quality and pentosan content may not be the same between the flours. Nevertheless, normalising the protein and water contents of the doughs would increase the reliance of the resultant baking quality on the gas bubble formation and stability during mixing and proving.

## 5 Discussion

Lipids from 2013 flour had the greatest impact on the interfacial properties and foaming of DL and the baking quality of the flour compared to flours from 2011 to 2012.

Surface dilatational rheology has showed that both lipids and proteins can adsorb at the air-water interface of DL and, typically, that lipids dominated the interface at higher concentrations of DL while proteins had more influence when the DL was diluted (Salt et al., 2006). Differences in the surface elasticity - surface pressure relationship (Fig. 2) were observed for undiluted 2013 DL, compared to 2011 and 2012 DLs, indicating that this sample had a different surface composition to the 2011 and 2012 DLs. The surface pressure of the 2013 DL was lower than the other two samples, which could indicate that the protein components could be having an influence (Salt et al., 2006). However, all samples displayed high surface pressure values, typical of interfaces dominated by surfactants or polar lipids. In addition, the surface rheology results (Fig. 1) showed that all samples behaved very similarly, displaying a weak elastic interface, and showed a maximum in surface elasticity when diluted. This maximum was shown to be due to the increased adsorption of the protein component (Salt et al., 2006), and the subsequent reduction in surface elasticity at higher DL concentrations shows that the interfaces are becoming increasingly dominated by the lipid component. Dough liquors and foams were therefore analysed to determine differences in lipid content and composition in to order explain differences in functionality at the gas bubble surface.

Enrichment of polar lipids (DGDG, MGDG, and PC) was observed in DL, with the highest concentration being observed in the 2013 DL and Foam (Fig. 4). These lipids were also present at higher proportions in the 2013 foam than in 2011 and 2012 foams, and could have contributed towards the increased stability of 2013 foam. Polar lipids, particularly galactolipids, have a large, non-ionic head group and are able to diffuse rapidly to the air-water interface making them suitable for stabilising foams and gas bubbles in bread dough (Gerits et al., 2014, Sroan and MacRitchie, 2009). However, 2013 DL and foam had higher protein contents, than 2011 and 2012 samples. Although this could also have contributed towards the improved foam stability, the weak surface elasticity values (Fig. 2) suggest that it was more likely that lipids were the main contributors to foam stability. Enrichment of total FFA and DAG was also observed in the 2011 and 2012 DLs and DL foams; and to a lesser effect for the 2013 DL foam. However, no significant enrichment in individual molecular FFA species differing in chain length or saturation was observed. The amount of FFA and DAG could have affected the foam stability, particularly that of the 2011 foam which contained higher levels of FFA and DAG. Free fatty acids are poorly soluble, have a small head group and are unable to diffuse quickly to the air-water interface, resulting in poor foam-stabilising characteristics. Also, small amounts of FFA are known to be detrimental to foam stability (Pareyt et al., 2011, Wilde et al., 2003), due to FFA using a foam breaking mechanism (Wilde, 2000), causing a rapid loss of foam structure, and therefore making them undesirable for breadmaking. The lower levels of polar lipids in the 2011 and 2012 foam, compared to levels in 2013 foam, meant that the overall stability of the foam was poorer than 2013 foam, and therefore its improved foam stability suggests that the higher levels of observed polar lipids are capable of stabilising foams, and therefore the gas bubble network in bread dough (MacRitchie and Gras, 1973, Sroan and MacRitchie, 2009). Even though the 2013 foam had high levels of neutral lipids, similar to the 2011 foam (Fig. 4), the much higher concentrations of polar lipids in the 2013 foam has probably counteracted the detrimental effect on foam stability. Flours from 2012 to 2013 were also used for test baking to determine the effects of differences in dough lipids on breadmaking quality. It was clear that the flour from 2013 gave the highest loaf volume, with a slightly larger gas bubble diameter (Fig. 7). The lipid analysis (Fig. 4), PCA data (Fig. 6) and the interfacial properties of DL (Fig. 1, Fig. 2) suggest that the polar lipids were dominating the interfacial properties. In addition, the dough recipes were adjusted to account for protein content and water holding capacity, which will increase the reliance for loaf volume on the stability of the gas cells. Hence the results suggest that the intrinsic wheat lipids had some influence on the gas bubble stability of dough during proving and early stages of baking. This observation is consistent with the observed enrichment of polar lipids in the aqueous phase from the dough (Fig. 6), foam stability of DL (Fig. 3) and the previous observations on the effect of polar lipids on breadmaking quality (MacRitchie and Gras, 1973, Sroan and MacRitchie, 2009). Nevertheless, we cannot discount other factors such as gluten quality or pentosan content, that may have had additional effects on baking quality.

### 5.1 Conclusions

Increasing the content of intrinsic polar lipids and decreasing the content of neutral lipids would improve the breadmaking quality of wheat, by increasing the stability of the gas bubble network and preventing coalescence during proving. Our results provide direct evidence that polar lipids such as the galactolipids are enriched at the air-water interface, thus contributing towards improving gas bubble stability in bread dough. Increasing the endogenous polar lipids in wheat could also result in increases in health benefits, by allowing the reduction of salt and the amount of bakery fat or emulsifier used without compromising dough stability. However, this will only be possible if the polar lipids are present in sufficient quantities to stabilise the thin films that support the gas bubble network in dough.

## Funding

This work was supported by the BBSRC through the Crop Improvement Research Club [grant number BB/J019488/1].

## Appendix A Supplementary data

Crop Club Dough liquor Supplementary Info Crop Club Dough liquor Supplementary Info
