# Supplementary material for: Intrinsic wheat lipid composition effects the interfacial and foaming properties of dough liquor
Source: Food Hydrocoll. 2018 Feb;75:211–22. doi: 10.1016/j.foodhyd.2017.08.020 (PMC5646524; doi:10.1016/j.foodhyd.2017.08.020)
Supplement: Crop Club Dough liquor Supplementary Info [file mmc1.docx]

Salt L.J. et al. Intrinsic wheat lipid composition effects the interfacial and foaming properties of dough liquor
**Supplementary Information**


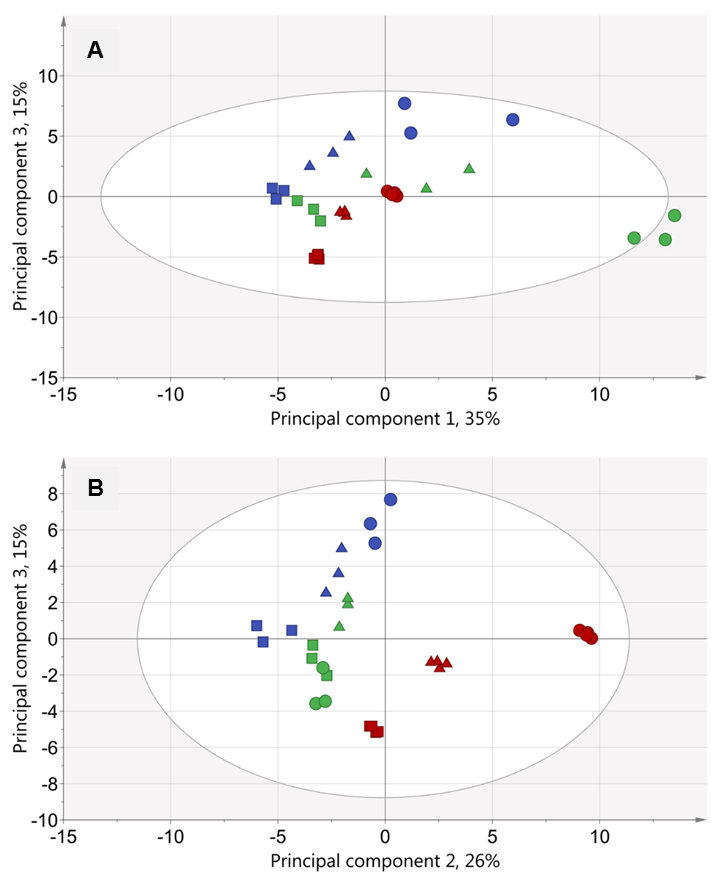


**Supplementary Figure S1:** Principal Component Analysis (PCA) on lipid composition of white flour (red), DL (green) and foam (blue) samples from 2011 (⯀), 2012 (▲) and 2013 (●). A: PCA scores plot showing PC1 (35 %) vs PC3 (15 %); B: PCA scores plot showing PC2 (26 %) vs PC3 (15 %).


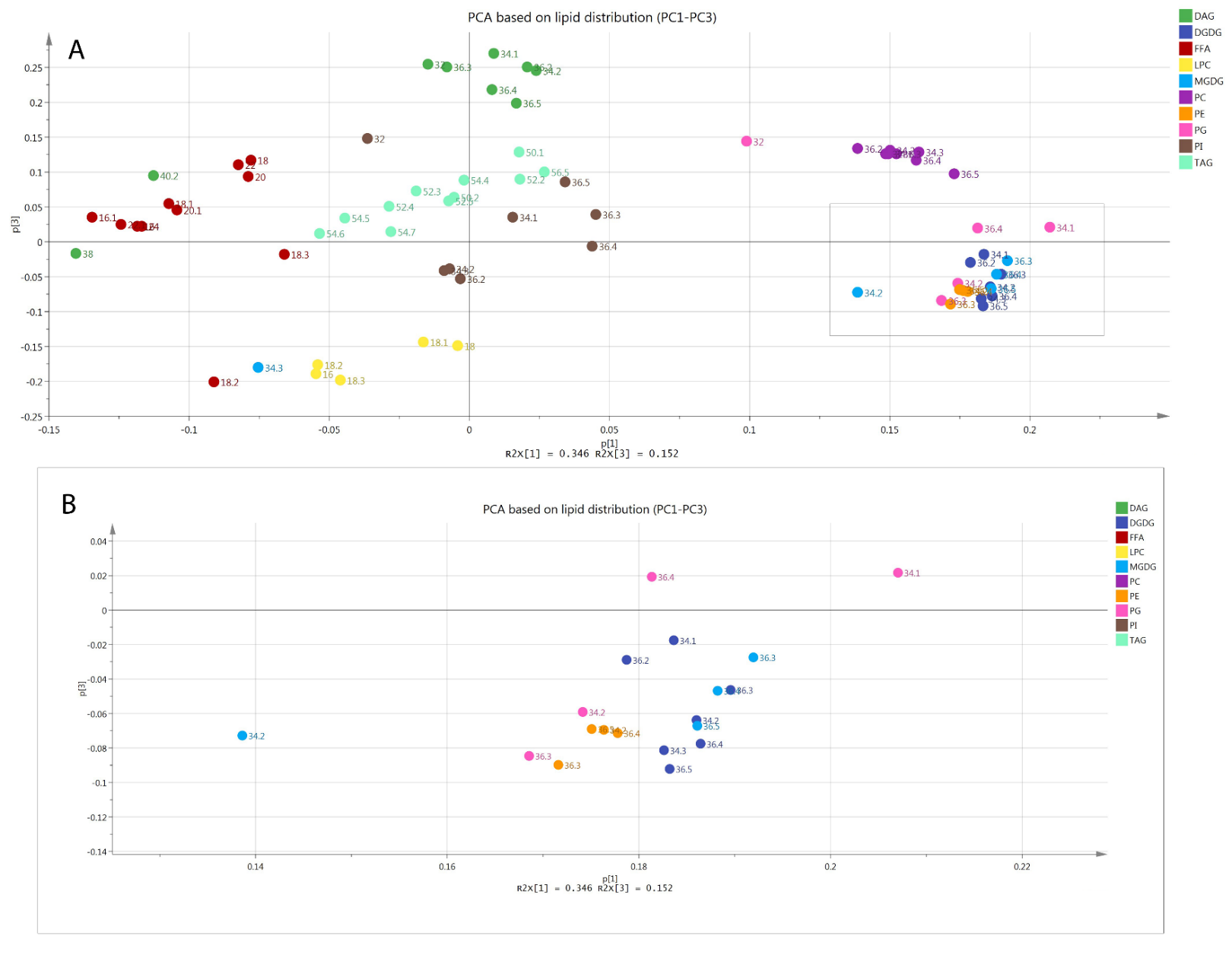
:

**Supplementary Figure S2:** Loading Scatter Plots from PCA on lipid composition of white flour, DL and Foam samples from three different years (2011,2012,2013). A, PCA loading scatter plot showing PC1 vs PC3. B, Detail of selected area (square) in plot A.


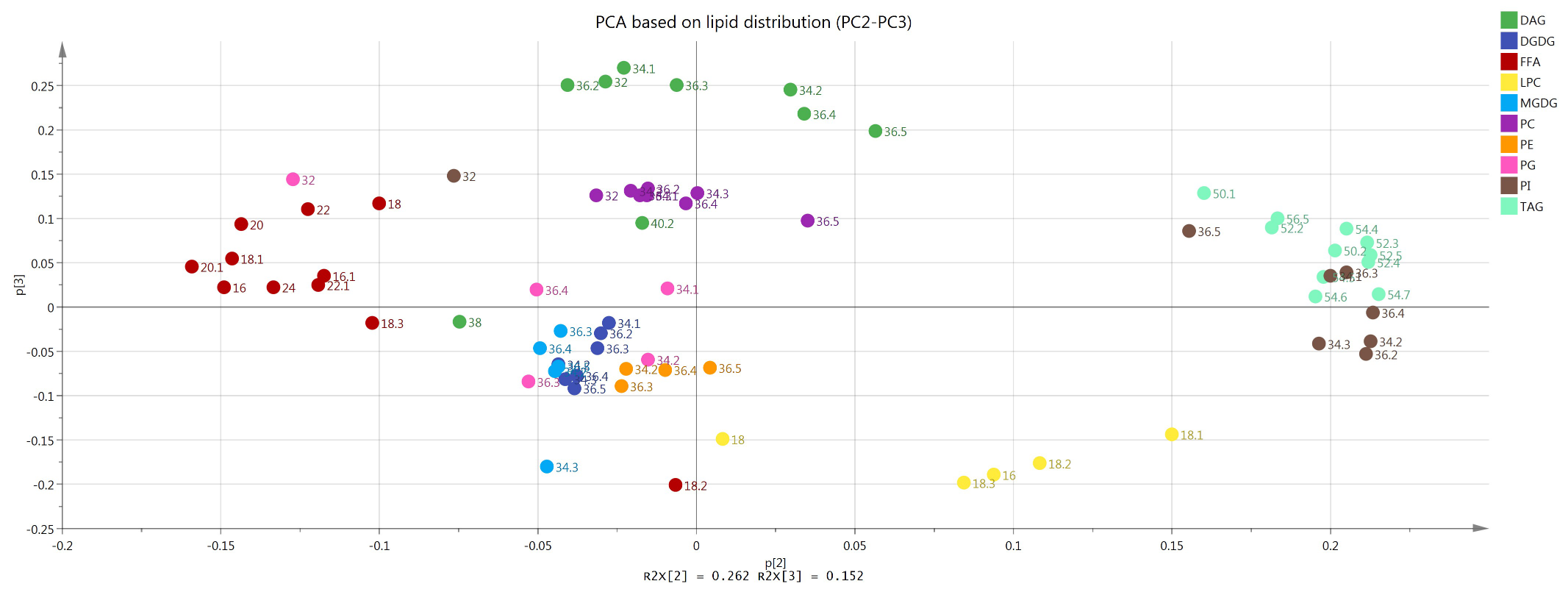


**Supplementary Figure S3:** Loading Scatter Plots from PCA (PC2 and PC3) on lipid composition of white flour, DL and Foam samples from three different years (2011,2012,2013).

**Supplementary Table S1.** Total lipid composition (nanomol/g flour) of White Flours, Dough Liquor and Foam from 2011,2012 and 2013. Lipid class and molecular species are indicated. Mean values and SE of at least three independent samples analysed via ESI-MS/MS (see material and method section) are shown.

**Supplementary Table S1.** (Contd.)


**Supplementary Table S1.** (Contd.)


**Supplementary Table S1.** (Contd.)


**Supplementary Table S1.** (Contd.)

**Supplementary Table S1.** (Contd.)
